# Cleavage of Histone 3 by Cathepsin D in the Involuting Mammary Gland

**DOI:** 10.1371/journal.pone.0103230

**Published:** 2014-07-23

**Authors:** Zhila Khalkhali-Ellis, William Goossens, Naira V. Margaryan, Mary J. C. Hendrix

**Affiliations:** 1 Cancer Biology and Epigenomics, Stanley Manne Children’s Research Institute, Chicago, Illinois, United States of America; 2 Robert H. Lurie Comprehensive Cancer Center, Northwestern University Feinberg School of Medicine, Chicago, Illinois, United States of America; King’s College London, United Kingdom

## Abstract

The post-lactational regression of mammary gland is a complex multi-step process designed to conserve the biological function of the gland for next pregnancy. This developmental stage is a biological intrigue with great relevance to breast cancer research, and thus has been the subject of intensive scrutiny. Multipronged studies (microarray, proteomics profiling, animal knock-out models) have provided a repertoire of genes critical to involution. However, the caveat of these approaches remains in their failure to reveal post-translational modification(s), an emerging and critical aspect of gene regulation in developmental processes and mammary gland remodeling. The massive surge in the lysosomal enzymes concurrent with the onset of involution has been known for decades, and considered essential for “clearance” purposes. However, functional significance of these enzymes in diverse biological processes distinct from their proteolytic activity is just emerging. Studies from our laboratory had indicated specific post-translational modifications of the aspartyl endopeptidase Cathepsin D (CatD) at distinct stages mammary gland development. This study addresses the biological significance of these modifications in the involution process, and reveals that post-translational modifications drive CatD into the nucleus to cleave Histone 3. The cleavage of Histone 3 has been associated with cellular differentiation and could be critical instigator of involution process. From functional perspective, deregulated expression and increased secretion of CatD are associated with aggressive and metastatic phenotype of breast cancer. Thus unraveling CatD’s physiological functions in mammary gland development will bridge the present gap in understanding its pro-tumorigenic/metastatic functions, and assist in the generation of tailored therapeutic approaches.

## Introduction

In adult nulliparous females, the mammary gland is mostly populated by adipocytes with the embedded epithelial network [1], [2]. Gestation initiates massive proliferation of the progenitor cells to form lobuloalveolar structures which will ultimately differentiate to milk secreting glandular epithelium upon parturition [3]–[5]. Cessation of suckling triggers a drop in lactogenic hormones and heralds the necessity for the involution. The involution process occurs in two stages [6]: In the first stage (reversible, lasting ∼48 h), despite the abundant alveolar cell death there is no remodeling of the glandular structure, this permits the continuance of secretory function if the suckling is resumed. In the second phase (non-reversible), the superfluous lobuloalveolar cells, their supporting matrix and accumulated milk are cleared by the combined action of lysosomal enzymes and matrix metalloproteinases, and the gland resumes an almost pre-gestation status [7]. Extensive efforts and multiple approaches including gene expression, proteomic profiles and animal knock-out models have identified genes critical to different stages of mammary gland development [8]. Notably, the knock-out models of genes critical for involution [9]–[16] have revealed delayed involution but none have actually stopped the process.

An undisputable attribute of involution is the significant induction of many proteolytic enzymes, specifically the lysosomal hydrolases [17]. Cathepsins B, D and L are elevated at the reversible stage of involution and remain high until 96 hrs post-weaning [17]–[20]. From functional perspective, this massive surge in activated enzymes is required for the clearance and remodeling of the redundant glandular structures. However, studies in the past decades have uncovered diverse and novel biological functions for these proteases [21], [22]. Specifically, recent exposé of their adipogenic effects [23]–[25] depict functional significance far beyond their conventional proteolytic properties.

The significance of post-trasnslational modification(s) of genes in developmental processes is just emerging. Studies from our laboratory were among the first to indicate the plasticity of mammary epithelium with respect to Cathepsin D (CatD) production, post-translational modification and activity [26]. Specifically, at the reversible phase of involution, CatD’s cleavage does not proceed beyond the generation of the single chain active enzyme [26]. This is concomitant with its Tyrosine nitration reported by Zaragoza and colleagues [27]. These precise and timely post-translational modifications prompted us to speculate on CatD’s significance in the involution process and re-population of the mammary tissue with adipocytes.

We employed an *in vitro* approach and treated normal mammary epithelial cells with CatD purified from involuting or lactating mouse mammary tissue. This approach exploited the capacity of mammary epithelial cells to capture CatD from the extracellular milieu (most probably via receptor-mediated endocytosis, [28]). Morphological and protein profiling analysis were employed to assess the differential effects of involution-derived CatD. The *in vitro* approach was further corroborated by an *in situ* approach using mammary tissue from different developmental stages, and defined a critical and previously unidentified function for CatD in mammary gland involution.

## Experimental Procedure

### Animals and Ethics Statement

Female C57BL mice (Harlan, Indianapolis, IN) were used at the following stages of development: lactating (3, and 7 days of lactation), post-lactation/involution (1, 2, 3, 4, and 7 days after the removal of pups on day 15^th^ of lactation). The animals were euthanized with ketamine and xylazine (80 mg/kg +10 mg/kg, ip) and sacrificed by cervical dislocation under deep anesthesia. Pectoral and inguinal groups of mammary glands were removed, and immediately frozen in liquid nitrogen for later use, or were fixed in 10% neutral buffered formalin, processed in a tissue processor and embedded in paraffin for immunoflourescence analysis. Six sets of mice at each stage of development were employed in these studies.

### Ethics Statement

All the animal protocols were reviewed and approved by Institutional Animal Care and Use Committee of Ann and Robert H. Lurie Children’s Hospital Research Center and Northwestern University Feinberg School of Medicine, which is AALAC accredited.

### Cell Culture

Normal human mammary epithelial cells HMEpC (Cell Applications Inc, San Diego, CA), were maintained in defined mammary epithelial cell medium provided by the company. Cultures were determined to be *mycoplasma* free using the GeneProbe rapid detection system.

### Tissue Extraction and Western Blot Analysis

The mammary tissues (inguinal glands) were removed, chilled in liquid nitrogen and were either used immediately or kept frozen at −80°C degrees for later use. The frozen tissue was pulverized in pre-chilled mortar and pestle and homogenized in buffer A (10 mM HEPES buffer pH 7.9 containing 10 mM NaCl, 1 mM DTT, 10% glycerol, 15 mM MgCl_2_, 0.2 mM EDTA, 0.1% NP40, protease/phosphatase inhibitor cocktail and pepstatin [1.2 µM]). The homogenate was subjected to 3 freeze-thaw cycles, passed several times through a 21-gauge needle and centrifuged (4500 rpm, 10 min) to yield a post-nuclear cytosolic fraction which was used for recovery and purification of CatD from different fractions (see below). The pellet was dispersed, washed repeatedly in buffer A, followed by incubation in buffer B (buffer A+500 mM NaCl and protease/phosphatase inhibitor cocktail and pepstatin [1.8 µM]) for 30 min on ice. Nuclear extract was obtained by centrifugation of the homogenate at 10.000×g for 25 min. The protein content of fractions was measured using BCA reagent. Equal amount of cellular protein were subjected to Western blot analysis using specific antibodies to human CatD (BD Transduction), mouse CatD (R&D System), H3 and K^23^H3 (Cell Signaling and Active Motif respectively), GAPDH and Lamin B (Santa Cruz Biotechnology), nitro-Tyrosine and Acid sphingomyelinase (Abcam). The HRP-labeled secondary antibodies were anti-goat (R&D Systems), anti-mouse and anti-rabbit (Bio Rad). The reaction products were visualized using the enhanced chemiluminescent kit (GE Healthcare). For equal loading and quality control of Western blots GAPDH and Lamin B were employed for HMEpCs and Acid sphingomyelinase and Lamin B for mouse mammary tissue extracts.

### Pepstatin Agarose Chromatography

CatD from post nuclear cytosolic fractions of mouse mammary gland at different stages of development was purified to homogeneity by three step purification: pepstatin agarose (Sigma) column chromatography at pH 5.5, followed by DEAE and Sephadex 75 column chromatography using well established protocols [29]. The purified products (from here onward referred to as mCatD) were applied to SDS-PAGE and probed by Western blot analysis. The purified mCatD preparations were diluted in growth medium at 2 µg/ml and added to HMEpC cultures. Purified mCatD was collected from 5 different sets of mice at specific developmental stages noted in the text and used in independent experiments.

### mCatD Treatment of Normal Mammary Epithelial Cells

Normal mammary epithelial cells grown either in 16 well chambered coverglass (Grace Bio-Labs, Inc., Oregon), or on Millicell culture inserts (Millipore) and allowed to polarize were treated with purified mCatD from different developmental stages at 2 µg/ml for 4–7 days. Cultures were monitored for morphological changes, and media was replaced every other day. At the end of culture period, cells were either fixed with ice cold Methanol for phase contrast and/or confocal microscopy or were used to make cytosolic and nuclear extracts.

### Alexa Fluor Labeling of Purified mCatD

In some experiments, in order to distinguish the endogenous CatD from the administered mCatD, the latter was labeled with Alexa Fluor 594 using Microscale Protein Labeling Kit (Molecular Probes, Invitrogen) and according to the manufactures specification. This labeled product was utilized as described above.

### Immunofluorescence Staining and Confocal Microscopy

Expression of proteins of interest in cultures of HMEpC ±treatment and in the mouse mammary tissue at different stages of development was examined by immunofluorescence analyses of methanol fixed HMEpC cultures, or formalin fixed, paraffin embedded mouse mammary tissue. Sections (4 or 10 µm) were deparaffinized, subjected to citrate buffer (pH 6.0) water bath antigen retrieval and blocked in 5% FCS/PBS. The primary antibodies used were anti-mouse CatD (R&D Systems), anti-human CatD, β-catenin (BD Transduction), followed by treatment with appropriate fluorochrome labeled secondary antibodies (Molecular Probes, Invitrogen), and the nucleus was stained with DAPI. The slides were cover-slipped with anti-fade mounting medium (Gelvatol) for confocal microscopy using the Zeiss LSM-510 META confocal laser scanning microscope equipped with ZEN software. Normal goat or rabbit IgG (at similar concentrations to the primary antibody) served as a negative control.

## Results

### Uptake of Purified mCatD by Normal Mammary Epithelial Cells

**Figure 1:**
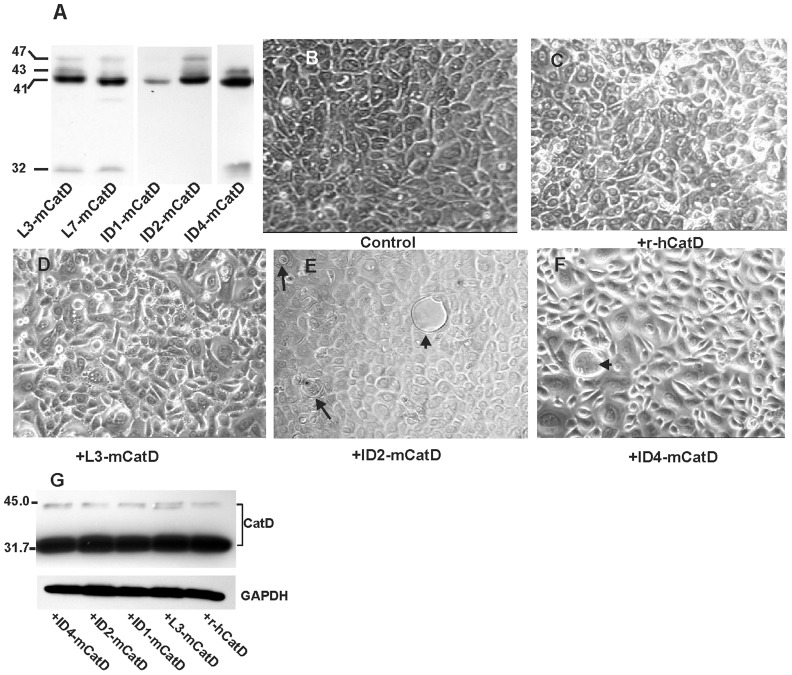
CatD purified from involuting mouse mammary tissue induces morphological changes in normal mammary epithelial cells. (A). SDS-PAGE (12.5% gel) and Western blot analysis of purified mCatD from different stages of development. (B–F). Phase contrast images of normal mammary epithelial cells following treatment with mCatD purified from distinct stages of mouse mammary gland development. Involution-derived mCatD induces the generation of few large cells with scant cytoplasm (arrowhead in E &F) in cultured epithelial cells. Recombinant hCatD and lactation-derived mCatD fail to induce comparable changes (C–D). The arrow in E points to cells presumably fusing. Original magnification:10×. (G). Western blot analysis of total cell lysates from treated cells indicated minimal effect of exogenously added mCatD on HMEpC’s endogenous CatD expression (probed by anti-human CatD, and seen as single chain ∼43 kDa and ∼32 kDa mature enzyme). GAPDH was used as loading control.

As a prelude to evaluating the functional significance of CatD associated with the involution stage, we purified CatD from the tissue extracts of the mouse mammary tissue (hereafter referred to as mCatD) at involution days 1, 2 and 4 (ID1, ID2 and ID4), and lactation days 3 and 7 (L3 and L7). A fraction of the purified mCatD was applied to SDS-PAGE and probed by Western blot analysis using anti-mouse CatD (Fig. 1A). This approach further supported our previous observations [26], highlighting differences in the apparent molecular mass and cleavage to mature enzyme at early involution stages (Fig. 1A, note the absence of mature 32 kDa form of the enzyme in ID1- and ID2- derived mCatD). Treatment of HMEpCs with mCatD from distinct stages of development provided the first indication of their differential effect. Notably, in cultures treated with involution-derived mCatD (ID2, or ID4) features indicative of cell fusion (or cells engulfing other cells) were apparent (Fig. 1E, arrows). Recombinant human CatD (r-hCatD) or lactation-derived-mCatD failed to promote such a process (Fig. 1C–D). By day 6 of treatment, some large cells with scant cytoplasm were noted in cultures treated with the ID2- and ID4-derived mCatD (Fig. 1 E–F, arrowhead). These cultures were moderately positive for Oil Red indicating the presence of lipid droplet (Fig. S1). The exogenously administered mCatD exerted no effect on the level and processing of endogenous CatD as determined by Western blot analysis of cell extracts using species-specific antibody (Fig. 1G).

**Figure 2:**
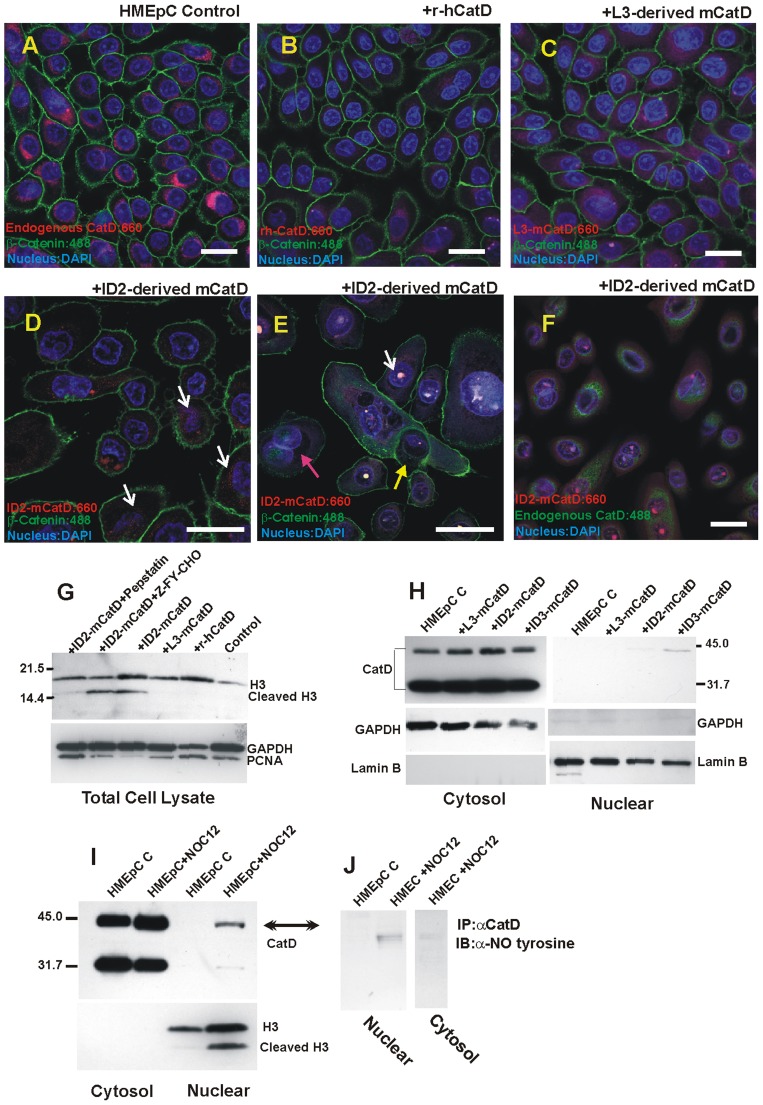
Confocal imaging and Western blot analysis of HMEpCs ± treatment with mCatD derived from lactation and involution stages. (A–F) Confocal images of HMEpCs ± treatment with Alexa 594 tagged mCatD preparations indicated involution-derived mCatD was transported mostly to the cytosol and often intensely localized to the nucleus (D & E, white arrow). It also promoted the process of entosis (cells engulfing other cells, D & E). Different stages of engulfment are captured in Fig. 2E (yellow arrow indicates an engulfed cell with intact membrane, the red arrow points to a cell with two nuclei). Occasionally, fragmenting nuclei of an engulfed cell could also be seen, Fig. S3). Endogenous hCatD is highlighted in (A) by anti-hCatD, followed by Alexa Fluor 660 secondary, red fluorescent in the image). Additionally, positional differences between endogenous CatD (green fluorescence) and ID2-derived mCatD (red fluorescence) are depicted in Fig. 2F. Please note a similar pattern of distribution for r-hCatD (B), and L3-derived mCatD (C) with the endogenous CatD (mostly lysosomal, A&F). β- Catenin is depicted by Alexa Fluor 488 (green fluorescent) and the nucleus is stained with DAPI. Original magnifications: A–C &F: 40x, D&E:100x Images A–F are also presented as split images in Fig. S2. Scale bar represents 20 µm. Total cell lysates (G) as well as cytosolic and nuclear fractions (H) of HMEpCs ± mCatD treatments were subjected to Western blot analysis to determine intracellular localization of mCatD compared to untreated control. Only involution-derived mCatD (from day two onwards) translocated into the nucleus, cleaved H3, the H3 cleavage was inhibited by pepstatin, but not by CatL inhibitor Z-FY-CHO. GAPDH and Lamin B were used as loading controls with PCNA depicting changes in the proliferation following the treatment. GAPDH and Lamin B were also used as a quality control to confirm the absence of contaminating cytosolic or nuclear proteins in the nuclear and cytosolic fractions respectively. (I). Cytosolic (20 µg) and nuclear fractions (60 µg) from HMEpCs ± NOC12 were subjected to SDS-PAGE (4–20% gel) and Western blot analysis to determine the effect of nitration on CatD processing and cellular distribution. (J). Cytosolic and nuclear associated CatD were immunoprecipitated and subjected to Western blot analysis using anti-nitro tyrosine antibody.

To further verify these observations and examine the intracellular localization of the administered mCatD compared to endogenous CatD, we tagged the mCatD preparations with Alexa Fluor 594 and treated HMEpCs (grown on 16 well chambered coverglass) with these tagged mCatD preparations. The r-hCatD similarly tagged was used as control. Following 5 days treatment regimen, the cultures were fixed and mCatD’s intracellular distribution was examined by immunofluorescence confocal microscopy and compared with that of r-hCatD and the endogenous CatD. This approach verified the intake of purified mCatD preparations by HMEpCs, and revealed unexpected differences in the intracellular distribution of the administered ID2-derived mCatD compared to the endogenous CatD. Notably, endogenous CatD was mostly lysosomal (Fig. 2A and F), while ID2-derived mCatD was more dispersed in the cytosol and often intensely localized to the nucleus (Fig. 2D & E, white arrows, please see Fig. S2 for split images). Lactation-derived mCatD exhibited a clear vacuolar and some cytosolic distribution and no nuclear localization, while r-hCatD somewhat mimicked the endogenous CatD localization (Fig. 2C&B respectively). These experiments were repeated with mCatD preparations from 5 different sets of mice and representative figures are provided in Fig. 2.

The confocal microscopy approach also confirmed features indicative of cells “engulfing” or “invading” another cell in HMEpC cultures treated with ID2-derived mCatD (Fig. 2D–F). Figure 2E has captured two such events: one designated by yellow arrow depicts a viable cell with integral membrane engulfed by another; the second one indicated by red arrow reveals a cell with two intact nuclei but no visible membrane for the engulfed cell. Fragmenting nuclei were also observed in engulfed cells implying a possible mechanism of cell elimination by invasion of one cell into another (Fig. S3). Prior incubation of the involution-derived mCatD with the antibody to mouse CatD abolished the observed morphological changes (data not shown).

### CatD’s Nuclear Translocation is Associated with the Cleavage of Histone 3

The nuclear translocation of the administered ID2-derived mCatD was quite unexpected and led us to speculate that it must be accompanied with changes in Histone 3 protein (H3) [30]–[32]. By employing Western blot analysis of total cell lysates from treated cells we noted the appearance of anti-H3 reactive smaller fragment in ID2-derived mCatD treated cells (Fig. 2G). Incubation of ID2-derived mCatD with pepstatin (the aspartyl endopeptidase inhibitor) prior to addition to the cultures abolished the cleavage of H3 (Fig. 2G). The inhibitors of other Cathepsins, specifically Cathepsin L (CatL), which has been shown to cleave H3 [33], had minimal effect on the H3 cleavage (Fig. 2G). In addition, ID2-derived mCatD exerted anti-proliferative effect on HMEpCs, as indicated by reduced PCNA levels (Fig. 2G).

To demonstrate nuclear translocation of mCatD, cytosolic and nuclear fractions of the HMEpCs± treatment were prepared and subjected to SDS-PAGE and Western blot analysis. Nuclear fractions were probed with species-specific (anti-mouse) which indicated the presence of mCatD in the nuclear fraction of the HMEpCs treated with involution-derived but not lactation-derived mCatD preparations (Fig. 2H).

### CatD’s Nuclear Translocation is Mediated by its Post-translational Modification

Thus far, our data support the contention that the involution associated CatD is functionally distinct, demonstrated by the specific post-translational modifications of CatD as suckling ceases and the gland prepares for involution. Specifically, CatD is tyrosine nitrated [27] and its processing is limited to the generation of the single chain active enzyme (∼41 kDa, [26]). We speculated that these modifications could signal CatD’s nuclear import. We asked the question if *in vitro* tyrosine nitration could recapitulate similar changes in CatD processing and intracellular distribution. HMEpCs were treated with nitric oxide (NO) donor 1-hydroxy-2-oxo-3-(N-ethyl-2-aminoethyl)-ethyl-1-triazene (NOC12) and cytosolic and nuclear fractions were subjected to Western blot analysis. As indicated in Fig. 2I, the processing of CatD to the mature enzyme (∼32 Da) was considerably diminished, resulting in accumulation of the active single chain (∼41 kDa) concomitant with its translocation to the nucleus and the cleavage of H3 (Fig. 2I). Immunoprecipitation of nuclear (and cytosolic) CatD followed by Western blot analysis using anti-nitro tyrosine (Abcam) indicated the majority of nitrated CatD was localized to the nucleus, while cytosolic CatD had very low abundance of nitrated residues (Fig. 2J).

### CatD Nuclear Import and Cleavage of Histone 3 in the Involuting Mouse Mammary Gland

**Figure 3:**
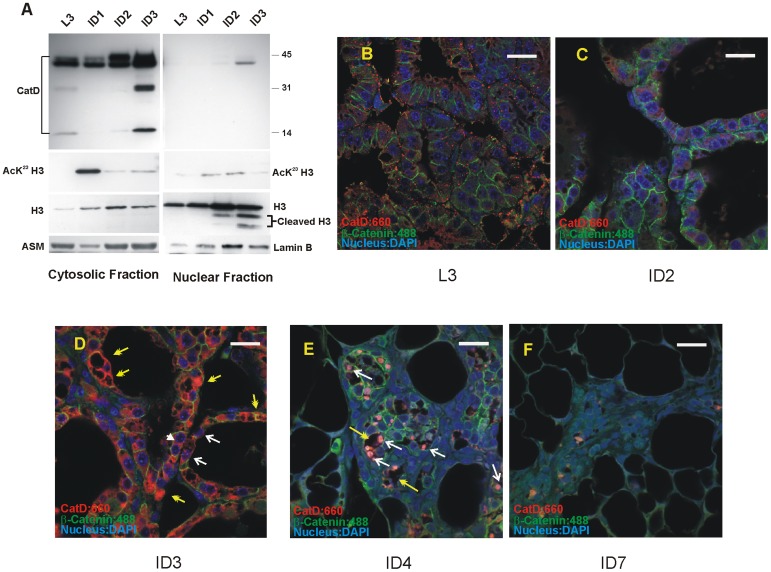
Onset of involution prompts nuclear translocation of CatD and cleavage of H3 in mouse mammary gland. (A). Western blot analysis of cytosolic and nuclear fractions from lactation and involution stages of mouse mammary gland reveals nuclear association of CatD at involution days 2 and 3. Cleavage of H3 occurs following the onset of involution. The cleavage of H3 may occur at multiple sites as indicated by the presence of several cleavage products of H3. H3 is also detected in the cytosolic fraction of the mammary gland at lactation and involution stages (soluble form) and is lysine ^23^ acetylated (AcK^23^H3) at involution day 1. The AcK^23^ could also be detected in the nuclear H3 but at a considerably lower abundance. (B–F). Immunofluorescence and confocal microscopy analysis of the formalin fixed, paraffin embedded mouse mammary tissue. Antibodies used were anti-mouse CatD and β-catenin followed by treatment with Alexa Fluor 488 secondary antibody for β-catenin (green) and Alexa Fluor 660 for mCatD (red). The nucleus was stained with DAPI. At involution day 3, CatD was detected in the nucleus (white arrows, Fig. 3D and specifically 3E), and sporadic multinucleated cells could be seen in the gland (white arrowhead, Fig. 3D). In addition, intense CatD immunostaining was noted in the structures reminiscent of phagosomes (Fig. 3E yellow arrows and Fig. S5). By day 7 of involution, the gland is mostly populated with adipocytes (Fig. 3F). Original magnifications: B–F 63x, scale bar represents 20 µm.

Our *in vitro* observations were further substantiated by our *in situ* approach using mammary tissue (at lactation and involution stages). CatD’s cellular distribution examined by Western blot analysis of post-nuclear cytosolic and nuclear extracts revealed nuclear presence of CatD on day 2–3 of involution (Fig. 3A). With CatD’s nuclear association, cleavage of H3 was also noted as early as day 2 involution (Fig. 3A compare with Fig. 2G & H). Additional H3 cleavage products (of less prevalence) were also detected in the nuclear fraction of involuting mammary gland (Fig. 3A). Of interest, H3 was also detected in the cytosolic fractions of the mammary gland which further corroborates the reported presence of soluble histones in the milk (and other body fluids, [34]). Notably, this soluble H3 was lysine^23^ acetylated predominantly at day 1 involution, the acetylated form decreased significantly by day 2 and was barely detectable at day 4 of involution (Fig. 3A). Probing the nuclear fractions for the presence of acetylated lysine^23^ indicated similar pattern of acetylation but lower abundance (Fig. 3A).

The intracellular distribution of CatD was further examined using immunoflourescence and confocal microscopy analysis of the formalin fixed, paraffin embedded mouse mammary tissue. This approach revealed intense and localized vacuolar association of CatD in day 3 lactation (Fig. 3B and Fig. S4A) compared to a muted and rather diffused cytoplasmic distribution in involution day 2 (Fig. 3C). Progression to day 3 and 4 involution was associated with elevated and intense cytoplasmic, and occasional nuclear association of CatD, specifically at involution day 4 (Fig. 3D&E, white arrows, Fig. S4C&D). By day 7 involution CatD level was considerably diminished and mostly detected in the areas still undergoing remodeling (Fig. 3F).

In addition, multinucleated cells (possibly representing entosis) were sporadically observed in the gland at involution day 3 (white arrow head, Fig. 3D and Fig. S4C).

### CatD Cleaves Histone 3 Between Lysine ^23^ and Alanine ^24^

**Figure 4:**
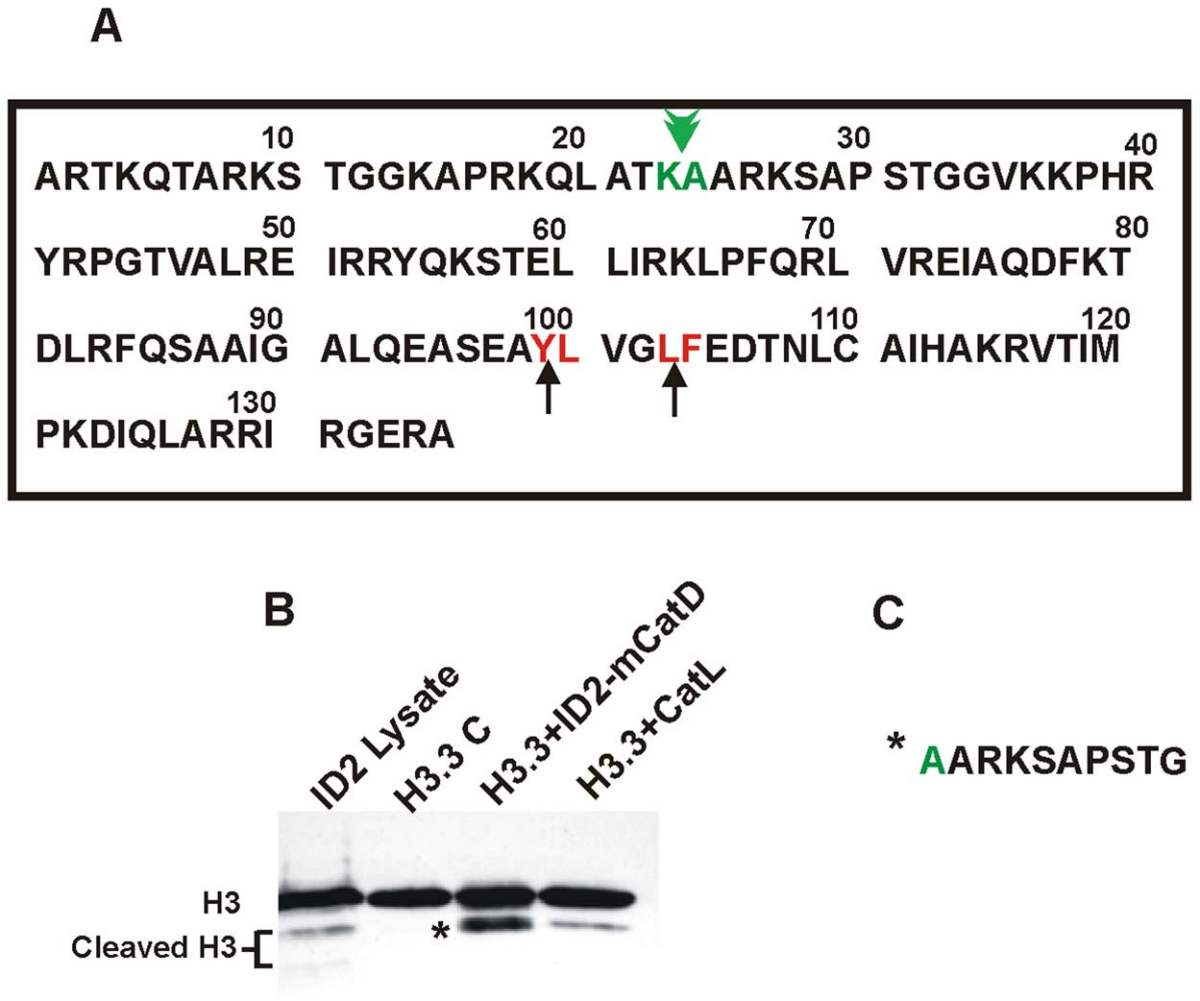
The observed H3 cleavage site (depicted by green color in A) by involution-derived mCatD. Theoretically predicted cleavage sites are also indicated (red in A). The in vitro cleavage of the recombinant H3.3 protein by ID2-derived mCatD and CatL is also indicated in Fig. 4B, with the total cell lysate of the mouse mammary gland at involution day 2 included as reference. The NH2 terminal sequence of H3 cleavage product (marked by *) is depicted in C.

The cleavage of H3 by CatD was tested *in vitro* using the recombinant H3.3 (BioLabs, Inc.) and involution day 2-derived mCatD with an enzyme/H3 ratio of 1/15 and at pH 6.5. As indicated in Fig. 4B, the apparent molecular mass of the cleaved product was similar to that observed in ID2 cell lysate. The cleaved fragment was subjected to Edman degradation and mass spectrometric analysis, which revealed the N-terminal sequence of the product to be A A R K S A P S T G (the cleavage occurring between lysine ^23^ and alanine ^24^ of the H3.3 N-terminus, indicated by arrowheads in Fig. 4A). This *in vitro* cleavage proceeded much faster at lower pH, and the recombinant human pro-CatD (r-hCatD, R&D Systems) failed to cleave H3 at a similar enzyme/H3 ratio without prior activation at low pH. However, much higher concentrations and longer incubation periods resulted in minor cleavage of H3 at similar pH (data not shown). Despite the presence of two theoretically favored cleavage sites, residues 99–100, tyrosine-leucine and 102–103, leucine-phenylalanine (marked by arrows in Fig. 4A), H3 was preferentially cleaved by CatD between lysine ^23^ and alanine ^24^. Recombinant CatL which has been shown to cleave H3 between alanine ^21^ and threonine ^22^ (as well as several other sites between amino acids 21 to 28 of H3) [33] also cleaved H3 generating a fragment with apparent molecular mass very close to that generated by ID2-derived CatD (Fig. 4B).

## Discussion

Previous studies from our laboratory and others had indicated unique post-translational modifications in CatD upon cessation of suckling [26], [27]. In the present work, the biological significance(s) of these modifications are addressed using both *in vitro* and *in situ* approaches. Purified CatD from mouse mammary tissue at different stages of development were tested for their biological effect(s) using an *in vitro* system of polarized normal mammary epithelial cells. This approach indicated the unique ability of involution-derived mCatD to promote the process of “entosis” (cell engulfing another cell), often leading to the generation of large cells with scant cytoplasm and moderately positive for Oil Red. Lactation-derived mCatD or r-hCatD failed to induce comparable changes.

Involution-derived mCatD has two distinct features; its processing is restricted to the generation of the single chain active enzyme [26] and is nitrated on tyrosine^168^ [27], features which we could recapitulate using an *in vitro* nitration approach. In addition, nitration of the single chain mCatD was sufficient signal for its translocation into the nucleus.

Tyrosine nitration as a signal for nuclear import has not been reported before and could impact our understanding of the factors regulating nuclear trafficking of proteins. However, it is important to remember that tyrosine nitration can greatly affect protein activity [35]–[37]. Both induction and reduction in protein activity is noted in nitrated proteins and presumably depend on the protein structure and position of the nitrated tyrosine residue(s) [38], [39]. In this context, the dramatic increase in CatD enzymatic activity noted at the involution stage [17], [20], [26], [27] could also be the consequence of its nitration; specifically, *in vitro* nitration of r-hCatD is reported to increase its enzymatic activity [27].

The key event following CatD’s nuclear translocation was the proteolytic cleavage of H3 at its amino terminal tail. This was noted in our *in vitro* model with the purified involution-derived mCatD, and *in vivo* in the involuting mammary tissue. Similarly, our *in vitro* nitration approach supported the nuclear translocation of CatD and cleavage of H3. Notably, despite the presence of two theoretically favored CatD cleavage sites, residues 99–100, tyrosine-leucine and 102–103, leucine-phenylalanine (marked by arrow in Fig. 4A), H3 was preferentially cleaved by CatD between lysine^23^ and alanine^24^. Based on our *in situ* examination of mammary tissue extracts the “histone code” endorsing this cleavage was acetylated lysine^23^ which emerged prominently in the cytoplasm at the onset of involution and declined thereafter. Thus far the proteolytic cleavage of H3 has been reported by viral foot-and-mouth disease protease 3C (a cysteine-like protease) in cultured mammalian cells infected with the virus [40], cysteine protease CatL (in mouse embryonic stem cells undergoing differentiation [33], and by an unidentified serine protease (in yeast [41]). The cleavage site for these diverse enzymes was mapped to leucine^20^- alanine^21^ for protease 3C, alanine^21^- threonine^22^ for CatL and the serine protease (with further cleavages at lysine^27^-serine ^28^, and some other minor cleavages noted for CatL) and differed from that observed in our study. Clearly, H3 modifications or “histone code” [42]–[44] has to be the determining factor in the site-specific cleavage by these different enzymes. In this context, the lysine^23^ acetylation was found inhibitory for CatL-mediated cleavage of H3 [33]. Detail analysis of H3 modifications will extend our knowledge of “histone code” required for H3 cleavage by CatD and the specific inductive and/or suppressive effect(s) these histone modifications might have on CatD enzymatic activity. It is plausible to speculate that the distinct changes in cytoplasmic H3 on day 1 involution, followed by the cleavage of H3 in the nuclear compartment, are signal(s) for terminating lactation and initiation of the involution process and are currently being investigated in our laboratory.

Our novel finding of the involvement of CatD in the process of “entosis” was somewhat unexpected. The engulfed cells were often degraded by CatD, leading to the generation of larger cells with scant cytoplasm, presumably in preparation for adipogenesis. The capacity of mammary epithelial cells to phagocytose apoptotic bodies (observed during the involution process) is well documented [45], [46]. However, their aptitude to engulf viable cells has only been demonstrated in the absence of attachment to the extracellular matrix [47]. In this process which is termed “entosis”, the internalized cells are either degraded by lysosomal enzymes (mainly Cathepsin B) or released. It is noteworthy that our experiments were performed with cells either attached to a membrane (polarized condition) or to a glass or plastic surface. Thus, it can be argued that the process of “entosis” could occur under anchorage-dependent or –independent conditions, via alternative signaling mechanisms and involve different Cathepsins.

Notably, based on our confocal microscopy, intense CatD staining was noted in certain structures which were prominent in the gland 48–96 hrs of involution. These structures were reminiscent of phagosomes and often contained nuclei (Fig. 3E and Fig. S4D and Fig. S5). It is noteworthy that similar structures have been reported by electron microscopic analysis of involuting gland as early as 1968 and were referred to as “cytosegrosome” [48]; however, the inclusion of nuclei in these structures was not reported.

In conclusion, our novel findings reveal for the first time previously unidentified function(s) of CatD in the involuting mammary gland. Based on our studies, the cessation of suckling results in the post-translational modifications of CatD which primes its nuclear translocation, cleavage of H3 at its –NH2 terminal between lysine^23^ and alanine^24^. Initially, H3 is acetylated on lysine^23^ on day 1 involution and could be detected as soluble H3 in the cytosolic fraction of mammary tissue. It is likely that this post-translational modification or “histone code” is the signal for its cleavage by CatD and initiation of irreversible stage of involution. Clearly, this function of CatD is not limited to mammary gland, future studies would reveal the significance of this endo peptidase in other developmental processes and embryogenesis.

From functional perspective, CatD is critically involved in breast cancer progression and metastasis, deregulated synthesis and elevated secretion of CatD are hallmarks of cancer [49]–[51]. Thus unraveling CatD’s physiological functions during development will bridge the present gap in our understanding of its pro-tumorigenic/−metastatic functions, and assist in developing appropriately tailored cancer therapeutics.

## Supporting Information

Figure S1 **ID2-derived mCatD induces morphological changes in polarized normal mammary epithelial cells.** (A & B). Normal mammary epithelial cells were cultured on Millicell culture inserts (Millipore) and allowed to polarize prior to the treatment with ID2-derived mCatD. The cultures were then fixed, stained with DAPI followed by Oil Red staining. Phase contrast microscopy for Oil Red is depicted in (A) and reveals weak but distinct Oil Red positive cells (arrowheads). Complementary DAPI staining (B) indicates the majority of Oil Red positive cells have multiple nuclei (cells engulfed by other cells, yellow arrowhead). The bar represents 50 µm. Bar graphs depict % Oil red positive cells (C), and the number of multinucleated cells (D) in polarized HMEpCs treated with ID2-derived mCatD respectively. The mean values were calculated from five separate experiments and the standard error of the mean is given for each graph.
(TIF)

Figure S2 **Confocal images which were depicted as overlay in** **Fig. 2** **are presented as spilt images to demonstrate nuclear localization of CatD noted in the ID2-mCatD treated HMEpCs.** Purified m-CatD from lactation and involution stages and the r-hCatD were Alexa 594 labeled prior to treatment (red fluorescence in B–F). The green fluorescence in A–E reflects β-catenin. In images D–E nuclear association of 594-labeled CatD is evident (arrows). Image F depicts differential localization of administered 594-labeled ID2-derived mCatD and endogenous CatD (green fluorescence). Original magnifications: A–C and F 40x, D and E 100x.
(TIF)

Figure S3 **Confocal image of HMEpCs treated with Alexa 594 tagged ID2-derived mCatD, depicting the fragmenting nucleus of an engulfed cell.** Original magnification: 100xwith 2x zoom, ID2-derived mCatD: 660 (red), β-Catenin:488 (green) and nucleus:DAPI.
(TIF)

Figure S4 **Confocal images which were depicted as overlay in** **Fig. 3** **are presented as split images and represent lactation day 3 (A), involution days 2 (B), 3 (C) and 4(D).** Original magnification: 63x, Alexa 488: β-Catenin (green fluorescence), Alexa 660: mCatD (red fluorescence), and nucleus stained with DAPI. Arrows indicate nuclear association of CatD.
(TIF)

Figure S5 **Confocal image of a representative section from day 4 involution (ID4) is depicted to illustrate the “phagosomes” (yellow arrows).** These structures often contained nuclei and were intensely stained for CatD (white arrows). The boxed area contains multiple examples of “phagosomes” with distinct surrounding membrane. Original magnification:63×, Alexa 488: β-Catenin (green fluorescence), Alexa 660: mCatD (red fluorescence), and nucleus stained with DAPI.
(TIF)
