# Supplementary figures and images for: Cleavage of Histone 3 by Cathepsin D in the Involuting Mammary Gland
Source: PLoS One. 2014 Jul 23;9(7):e103230. doi: 10.1371/journal.pone.0103230 (PMC4108390; doi:10.1371/journal.pone.0103230)

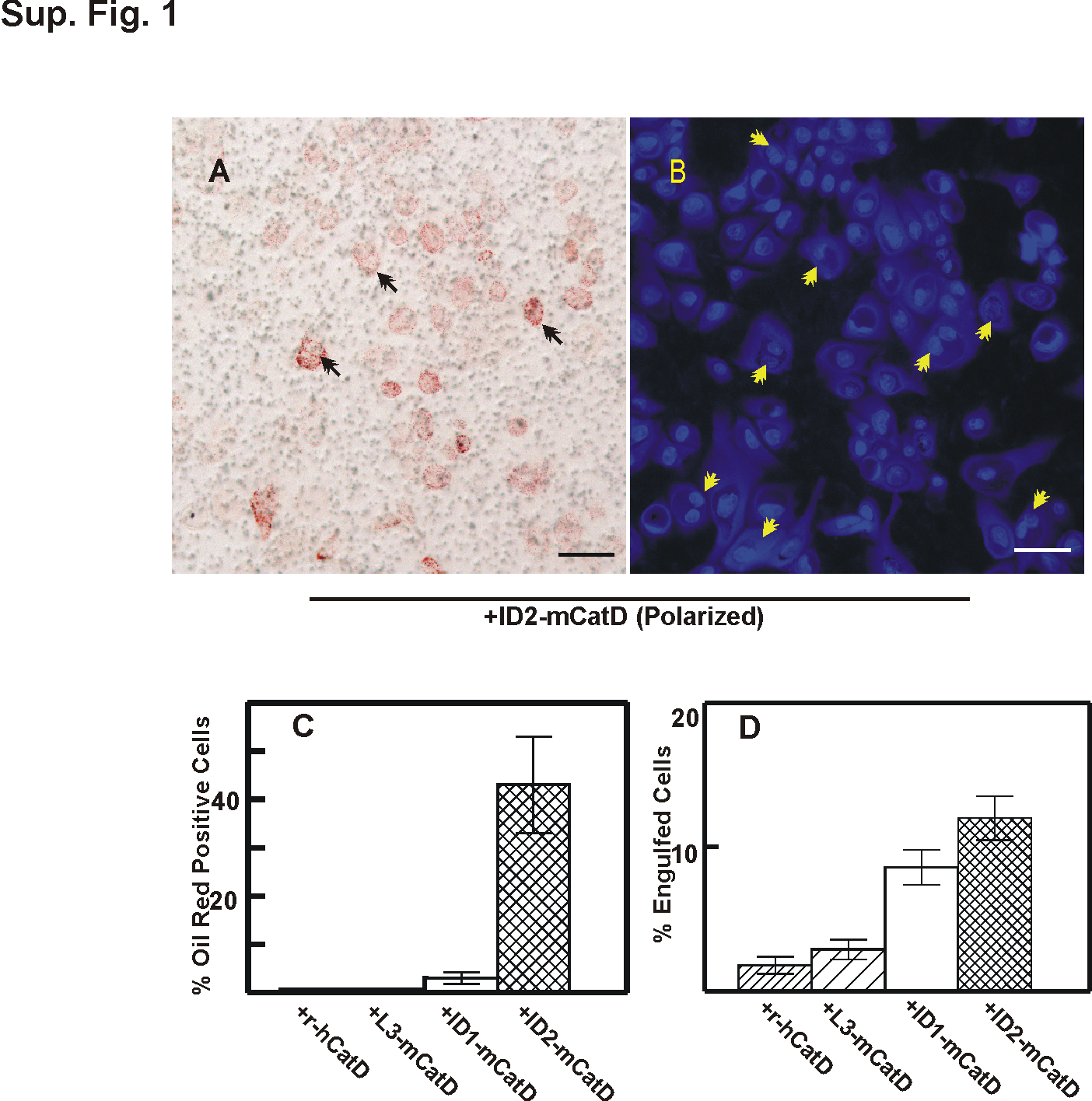

Supplement: Figure S1 — ID2-derived mCatD induces morphological changes in polarized normal mammary epithelial cells. (A & B). Normal mammary epithelial cells were cultured on Millicell culture inserts (Millipore) and allowed to polarize prior to the treatment with ID2-derived mCatD. The cultures were then fixed, stained with DAPI followed by Oil Red staining. Phase contrast microscopy for Oil Red is depicted in (A) and reveals weak but distinct Oil Red positive cells (arrowheads). Complementary DAPI staining (B) indicates the majority of Oil Red positive cells have multiple nuclei (cells engulfed by other cells, yellow arrowhead). The bar represents 50 µm. Bar graphs depict % Oil red positive cells (C), and the number of multinucleated cells (D) in polarized HMEpCs treated with ID2-derived mCatD respectively. The mean values were calculated from five separate experiments and the standard error of the mean is given for each graph. (TIF) [file pone.0103230.s001.tif]

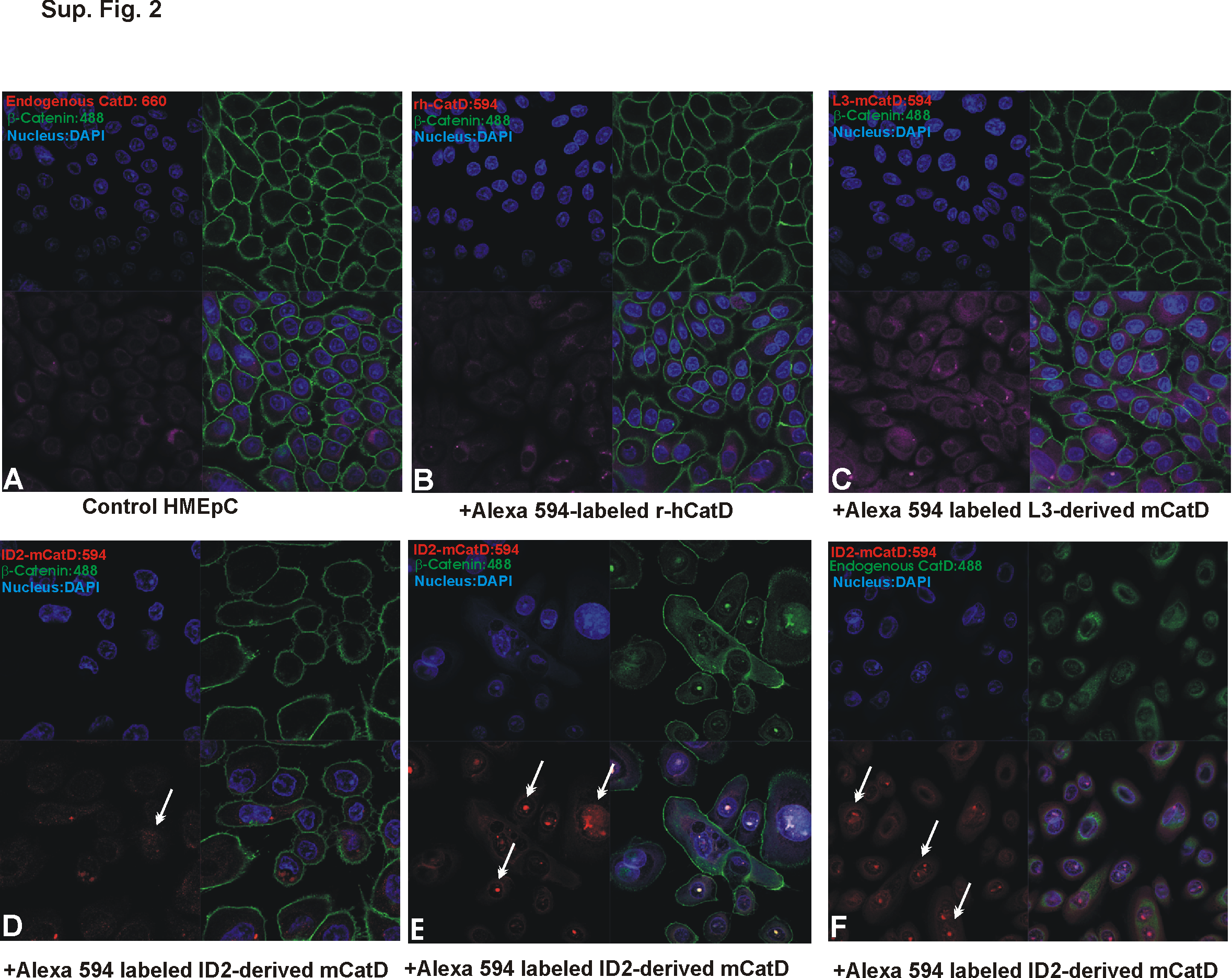

Supplement: Figure S2 — Confocal images which were depicted as overlay in Fig. 2 are presented as spilt images to demonstrate nuclear localization of CatD noted in the ID2-mCatD treated HMEpCs. Purified m-CatD from lactation and involution stages and the r-hCatD were Alexa 594 labeled prior to treatment (red fluorescence in B–F). The green fluorescence in A–E reflects β-catenin. In images D–E nuclear association of 594-labeled CatD is evident (arrows). Image F depicts differential localization of administered 594-labeled ID2-derived mCatD and endogenous CatD (green fluorescence). Original magnifications: A–C and F 40x, D and E 100x. (TIF) [file pone.0103230.s002.tif]

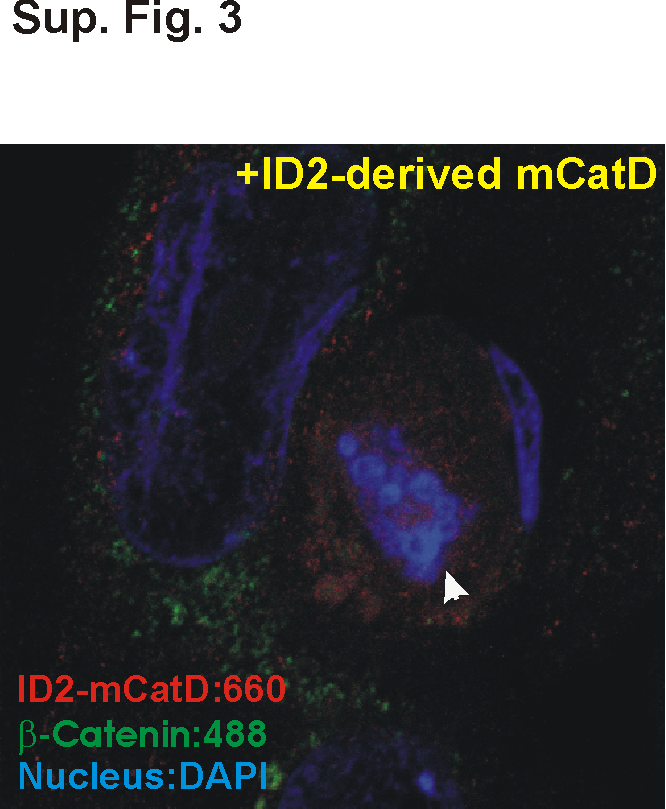

Supplement: Figure S3 — Confocal image of HMEpCs treated with Alexa 594 tagged ID2-derived mCatD, depicting the fragmenting nucleus of an engulfed cell. Original magnification: 100xwith 2x zoom, ID2-derived mCatD: 660 (red), β-Catenin:488 (green) and nucleus:DAPI. (TIF) [file pone.0103230.s003.tif]

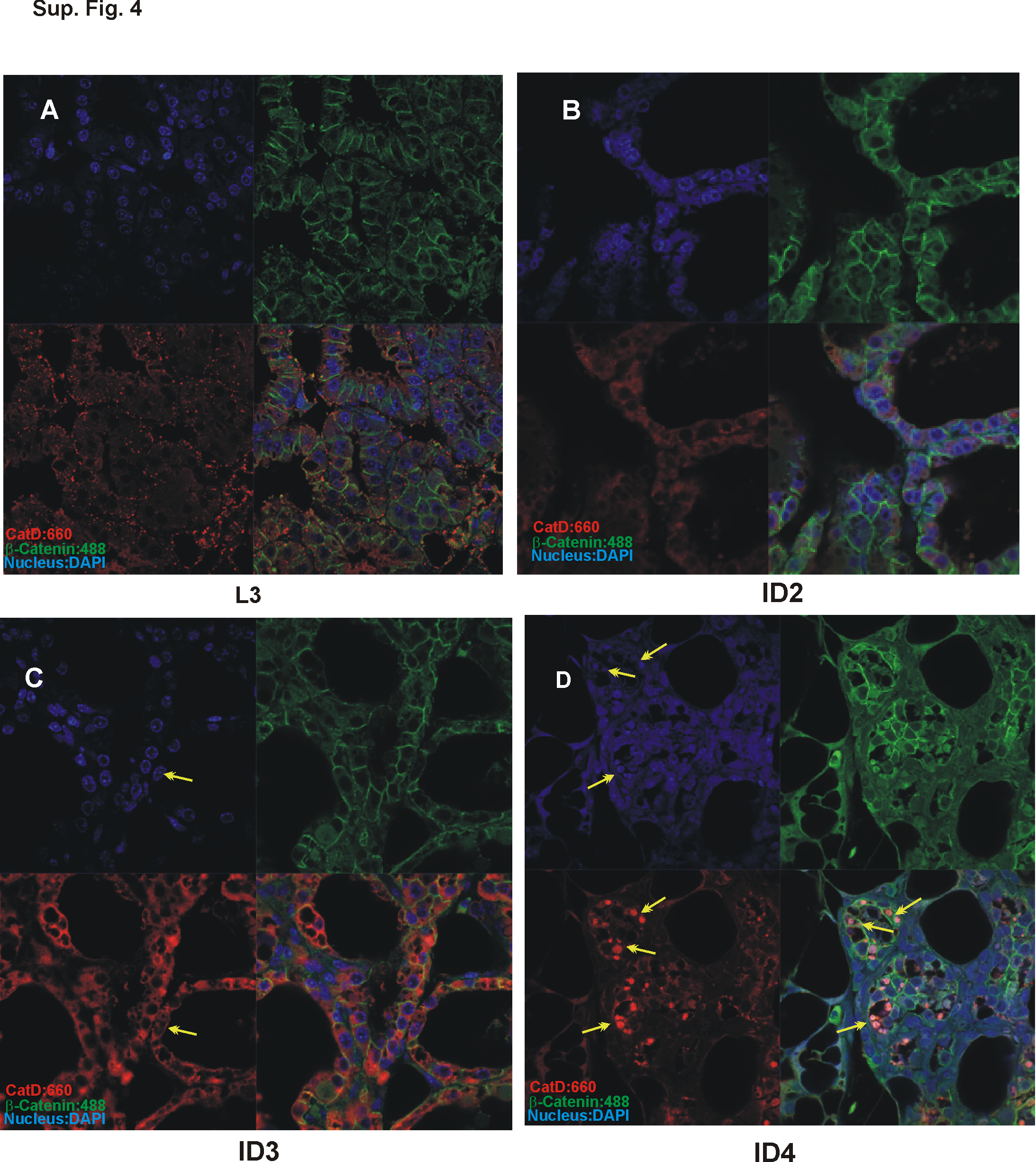

Supplement: Figure S4 — Confocal images which were depicted as overlay in Fig. 3 are presented as split images and represent lactation day 3 (A), involution days 2 (B), 3 (C) and 4(D). Original magnification: 63x, Alexa 488: β-Catenin (green fluorescence), Alexa 660: mCatD (red fluorescence), and nucleus stained with DAPI. Arrows indicate nuclear association of CatD. (TIF) [file pone.0103230.s004.tif]

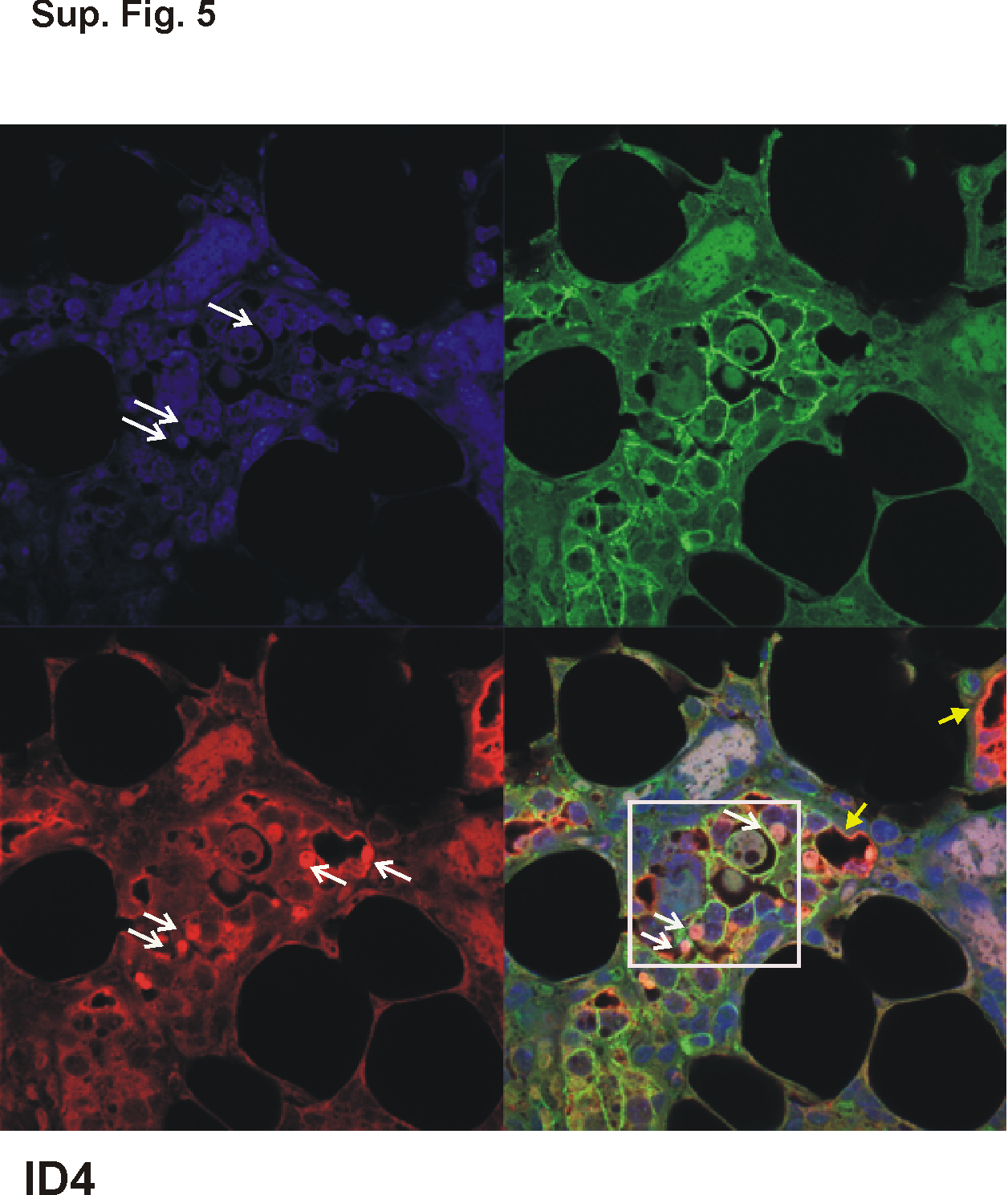

Supplement: Figure S5 — Confocal image of a representative section from day 4 involution (ID4) is depicted to illustrate the “phagosomes” (yellow arrows). These structures often contained nuclei and were intensely stained for CatD (white arrows). The boxed area contains multiple examples of “phagosomes” with distinct surrounding membrane. Original magnification:63×, Alexa 488: β-Catenin (green fluorescence), Alexa 660: mCatD (red fluorescence), and nucleus stained with DAPI. (TIF) [file pone.0103230.s005.tif]
